# Clinical Spectrum and Perspective in Bilateral Acute Retinal Necrosis: Systematic Review

**DOI:** 10.3390/medicina60111735

**Published:** 2024-10-23

**Authors:** Valeria Albano, Mariantonietta Di Brina, Maria Grazia Pignataro, Giacomo Scotti, Camilla Di Pardo, Giovanni Petruzzella, Antonio Salvelli, Rosanna Dammacco, Silvana Guerriero, Giovanni Alessio

**Affiliations:** Department of Basic Medical Sciences, Neurosciences and Sensory Organs, Eye Clinic, University Hospital Polyclinic of Bari, Piazza Giulio Cesare 11, 70124 Bari, Italy; mariantoniettadibrina@gmail.com (M.D.B.); mariagraziapignataro96@gmail.com (M.G.P.); giacomo.scotti@tiscali.it (G.S.); camilladipardo@gmail.com (C.D.P.); ivan.giovanni.petruzzella@gmail.com (G.P.); antoniosalvelli96@gmail.com (A.S.); r.dammacco@libero.it (R.D.); silvanaguerriero@gmail.com (S.G.); giovanni.alessio@uniba.it (G.A.)

**Keywords:** bilateral acute retinal necrosis, retinitis, retina, viral infection

## Abstract

Bilateral acute retinal necrosis (BARN) represents a broad ophthalmological field of severe retinal pathologies associated with poor visual prognosis and blindness. The purpose of this review is to examine the clinical spectrum in detail over the past few years, exploring laboratory and instrumental diagnosis, and providing useful and up-to-date guidance in this field. A systematic review of this field has been performed through the PRISMA guidelines, searching in the PUBMED database. Serological laboratory tests on blood or polymerase chain reaction (PCR) on aqueous humor or vitreous samples are crucial to identifying the underlying cause and choosing the timeliest strategic treatments. Often, the main cause remains herpesviruses, with Varicella zoster (VZV) predominating over the others. There are also other causes that one needs to be carefully aware of. Anatomical and functional recovery is unfavorable if they are not individuated in a quick time. Early diagnosis and timely treatment offer a better chance of visual improvement and the avoidance of complications. Complications worsen the visual prognosis over months and may require a surgical approach.

## 1. Introduction

Bilateral retinal necrosis (BARN) comprises a complex spectrum of ocular disorders characterized by the degeneration and death of retinal cells in both eyes [1]. It is not a single entity, but it is better defined as a syndrome [2]. This condition, although relatively rare, has attracted increasing interest in the scientific community due to its unique clinical manifestations and associated diagnostic and therapeutic challenges. This review aims to provide a comprehensive overview of the current knowledge of BARN, diagnostic methodologies, and available treatment options. BARN can occur in a variety of clinical settings, including those related to systemic, infectious, or autoimmune diseases. Understanding the underlying pathogenesis of these retinal necroses is crucial for timely diagnosis and targeted treatment [3].

Acute retinal necrosis is common due to retinal infection characterized by retinal vasculitis, anterior chamber, and vitreous inflammation with evolution to retinal necrosis and retinal detachment [4,5].

In 1994, a classification of acute retinal necrosis was introduced by the Executive Committee of the American Uveitis, on the basis of the following clinical features: 1. one or more foci of retinal necrosis with discrete borders located in the periphery, 2. rapid progression in the absence of antiviral therapy, 3. circumferential progressions, 4. evidence of occlusive vasculopathy with arteriolar involvement, and 5. significant inflammatory reaction of the vitreous chamber and anterior chamber [6].

The first cases of bilateral acute retinal necrosis (BARN) were described by Willerson in 1977, and later the term BARN was introduced in 1978 by Young and Bird [7]. It can affect both immunocompetent and immunocompromised individuals of both sexes and all ages [8].

In most cases, a viral cause was confirmed upon seeing by investigative methods such as polymerase chain reaction (PCR) on aqueous humor and vitreous samples [9]. The most involved viral agent seems to be Varicella zoster (VZV), followed by Herpes simplex 1 and 2 (HSV1 and HSV2), Cytomegalovirus (CMV), and Epstein–Barr virus (EBV) [10]. Retinitis in most cases begins in the retinal periphery and has centripetal involvement, except for EBV-related forms which predominantly affect the macular region with significant arterioles occlusion [11]. It is important to start antiviral therapy early to reduce the risk of contralateral eye involvement [12].

BARN does not always have a viral infectious cause. BARN represents a more heterogeneous group of bilateral retinal diseases, which can also be caused by bacterial infections, fungal infections, autoimmune diseases, and drug toxicity, such as antineoplastic drugs [13]. This is important because the treatment will depend on the underlying cause [14].

The mechanisms responsible for the involvement of the contralateral eye have not yet been fully elucidated. Based on animal models, it is thought that in the case of a virus agent, it may migrate into the contralateral eye through the nerve pathways of the nerve and optic chiasma [15]. However, the altered balance between viral replicative activity and the host immune response seems to have a crucial role. Cases in the literature have demonstrated the link between viral reactivation and immunodepression associated with conditions such as diabetes, chronic corticosteroid use, and immunodepression resulting from the chronic use of immunodepression biological drugs [16]. Other mechanisms other than the virus that lead to retinal damage are not yet well known.

BARN is often associated with poor visual prognosis and blindness. The early identification of BARN is crucial to prevent irreversible retinal damage and preserve visual function. In this context, recent advances in diagnostic techniques, including high-resolution imaging and genetic analysis, which have contributed significantly to diagnostic accuracy in this field, will be discussed.

Furthermore, the current treatment options available for BARN, including pharmacological approaches and surgical interventions, will be explored. The efficacy of these treatments will be evaluated based on the latest scientific evidence, with a focus on long-term results and possible side effects.

This article aims to provide an in-depth and up-to-date overview of bilateral retinal necrosis, offering a practical guide for ocular health professionals in the early recognition, accurate diagnosis, and optimal treatment of this complex disorder.

## 2. Methods

**Figure 1:**
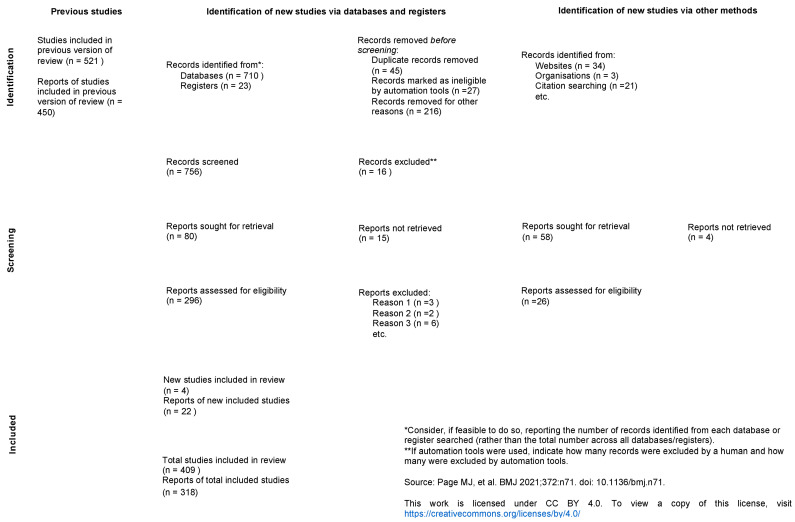
PRISMA 2020 flow chart for updated systematic reviews which included searches of databases, registers, and other sources. Page M J, McKenzie J E, Bossuyt P M, Boutron I, Hoffmann T C, Mulrow C D et al. The PRISMA 2020 statement: an updated guideline for reporting systematic reviews BMJ 2021; 372: n71 doi:10.1136/bmj.n71 [17].

A scrupulous literature search was achieved through the PubMed database. The keywords used are as follows: “bilateral acute retinal necrosis”. Under these terms, the “acute retinal necrosis” was also included which we have decided to leave within. The systematic review was performed following the PRISMA 2020 guidelines [17] which included searches of databases, registers, and other sources (Figure 1). We included all searches that were set from 1978 to June 2024 in publishing, focusing mainly on the last decade, from 2010 onwards.

We collected 409 articles in total. Observational studies, multicenter studies, case series, case reports, reviews, books, and experimental studies were included in the review. All the reviewed articles are strictly in the English language. Other studies that did not respond to these criteria are excluded.

## 3. Results and Discussion

A total of 409 articles were reviewed from the literature, of which 277 were case reports, 41 case series (671 patients in total), 2 multicenter studies (50 patients in total), only 1 review, 2 books, and 15 experimental studies on lab rats. All 1013 cases of BARN were collected.

The age of the cases was variable, ranging from 5 to 76 years; also, preterm babies and neonates were described.

Frequently, it occurs in immunocompromised patients, but in immunocompetent subjects, it is also shown in the literature.

### 3.1. Pathogens and Other Causes

The common cause of BARN is *herpesviral* pathogens, and most frequently Varicella zoster (VZV) in 20.8% of the cases and Herpes simplex (HSV1 and HSV2) in 10.3% of the cases were reviewed. Especially in immunosuppressed patients, Cytomegalovirus (CMV) 3.3%, Epstein–Barr virus (EBV) (0.8%), and Herpesvirus6 HHV6 (0.2%) were also described [14,18,19,20]. Some reports reported Toxoplasmosis infection as an atypical presentation (0.7%), simulating acute retinal necrosis syndrome [21].

Other pathogens found were Pseudorabies Virus (PRV) (0.3%), Coronavirus (0.3%), BK Polyomavirus (0.1%), *Mycobacterium tuberculosis* (0.1%), *Aspergillus* (0.3%), and *Treponema pallidum* (0.4%) in the atypical form [22,23,24].

One singular case is caused by a nematode, *Angiostrongylus cantonensis* (0.1%) [25].

The cause of retinal necrosis is not always determinate, because in 18 cases PCR on humor aqueous or blood tests were negative. In these cases, empirical antiviral therapy was also initiated with improved outcomes [26].

Systemic immune or rheumatological diseases may also onset with retinal necrosis in the eye. The association with Behcet’s disease (0.3%), Wegener’s granulomatosis (0.1), Sarcoidosis (0.1), and ocular lymphoma (0.3%) has been reported [27,28,29].

Even in these cases, it is important to perform an ocular sample to identify the underlying cause and to set up a correct diagnosis and therapy. Finally, cases of iatrogenic retinal necrosis have also been described [30,31].

The number and types of articles in which bilateral acute retinal necrosis are cited, based on the causative pathogen or other agents, from searching in the literature are described in Table 1.

Table 1 displays the types of most causative agents and the number found in the literature. Moreover, it displays in which type of articles the bilateral acute retinal necrosis is cited on based on the causative agent. 

The main agents responsible for BARN, with typical features, were reported in Table 2.

Table 2 shows the main pathogens and other agents (in an alphabetic list) causative of bilateral acute retinal necrosis found in the PUBMED database, divided for immunocompromised (IC) and immunocompetent (IT) patients, correlating with some other clinical disorders or abuse conditions. The “1” value stands for association with a known clinical condition, “0” value stands for no association with any clinical condition.

One of the most widely used models for studying retinal necrosis involves the inoculation of herpes simplex virus into the eyes of animal models, such as mice and rabbits. Studies using these models have elucidated the role of viral reactivation and direct viral invasion in causing retinal cell death. Webre et al. [32] demonstrated that the inoculation of HSV-1 into the anterior chamber of rabbit and mouse eyes results in retinal necrosis, like that seen in humans. The study provided evidence that viral replication within retinal cells, combined with an overwhelming inflammatory response, leads to retinal destruction. HSV-induced necrosis models helped in identifying the mechanisms of retinal damage, including the disruption of the blood–retinal barrier and immune-mediated responses.

In immunocompromised individuals, especially those with HIV/AIDS or those receiving immunosuppressive therapy, CMV can cause aggressive retinal necrosis. Studies using animal models with induced immunosuppression have replicated these findings. Some studies, including work by Oh et al., have used mice with induced immunodeficiency to show how CMV infiltrates the retina, causing progressive necrosis. These models provided insights into the importance of both viral replication and immune system deficiency in the rapid progression of necrosis [33].

#### Signs, Complications, and Functional Outcomes

BARN are very aggressive uveitis with severe clinical manifestations, for which they need special clinical and surgical attention. They are characterized by retinal vasculitis with the involvement of the veins and arteries with the presence or absence of retinal hemorrhage. Clinically, they present as whitish or yellowish peripheral areas of multifocal necrotizing retinitis associated or not with intraretinal hemorrhages and papillary edema. The inflammation originates from the retinal periphery and progressively extends toward the posterior pole, potentially affecting the macula and optic nerve [34]. Other manifestations include vitritis, scleritis, flare in the anterior chamber, the presence of fine or mutton fat-like keratic precipitates, and increased intraocular pressure also with acute angle closure [35]. The most fearsome complication is the rhegmatogenous retinal detachment; in this case, the prognosis is very poor (best-corrected visual acuity, BCVA, Snellen 20/200 or less) [36]. Other complications include the development of macular holes, macular pucker, and ischemic optic nerve vasculopathy [8]. Clinically, the patient often complains of blurred vision, periorbital pain, reduced visual acuity, floaters, photophobia, pain with eye movements, redness, and foreign body sensation [5].

We have established that the BCVA does not depend on the initial underlying cause. Visual loss can be persistent if the diagnosis is not made in a timely manner [37]. An early start to diagnosis and the right therapy correlates with an improved visual recovery [38,39]. BCVA at the onset of symptoms and the complications that occur are reported in Table 3.

Table 3 shows the best-corrected visual acuity (BCVA) at the onset of symptoms and the complications related to BARN conditions, based on the causative pathogen or other agents.

### 3.2. Diagnosis

BARN is a form of inflammatory vasculitis whose diagnosis is primarily made through clinical examination. However, instrumental examinations such as fluorangiography (FA) and optical coherence tomography (OCT) are useful for defining the extension of the inflammatory process. In fact, there may often be discrepancies between the clinically observed picture and the fluoroangiographic (FA) picture [40]. FA allows the detection of the occlusion of arterial and venous vessels and nonperfused capillaries in early stages and venous and optic disk dye leakage, often edematous in late stages [41]. However, indocyanine green angiography has also been shown to be a useful tool to study the circulatory changes that characterize these uveitis forms.

Histopathological studies have demonstrated the presentation of inflammatory cells at the choroidal level in patients affected. Unlike the pattern of leakage observed with FA, in IA, the leakage is much less diffuse and affects only the areas of the retina intensely involved in the inflammatory process. Two cases have been described in which the dye leakage appears to start from the optic disk and follow the infero-temporal arcade, underscoring the inflammatory pattern and likely the type of migration of herpetic viruses involved in the viral forms of retinal necrosis [41]. Recently, the role of OCT and optical coherence tomography angiography (OCTA), and noninvasive examinations, especially useful in the monitoring and evolution of structural and vascular changes, is emerging.

OCT allows the detection of the presence of possible macular edema, hyperreflectivity of inner retinal layers, and loss of physiological architecture coincident with peripheral necrotic lesions. It has also been noted, moreover, that the marked thinning of the inner and outer retinal layers typical of necrotic areas, and the loss of the ellipsoid zone represents irreversible damage, which cannot be recovered by therapy [42]. Finally, OCTA does not allow us to highlight vascular leakage as in FA but helps us monitor microvascular changes in the superficial and deep capillary plexus at the macular level [43]. OCTA follow-up has also shown that the early initiation of antiviral therapy is able to restore capillary vascular flow in initially affected areas [44].

### 3.3. Therapeutic Strategies

#### Systemic Antiviral Therapy (Oral and Intravenous)

According to the acknowledged treatment protocol, an induction therapy consists of 10 mg/kg of systemic intravenous acyclovir three times a day or 3 g of oral valacyclovir in three divided doses for 7–10 consecutive days. The maintenance viral dose is 800 mg of oral acyclovir twice a day or 1000 mg of valacyclovir twice a day for at least 4 months. The renal function indices of patients on antiviral therapy should be checked frequently, adjusting dosages in cases of renal failure. It is recommended that the conditions of systemic immunodepression be recognized early on [45].

The corticosteroid therapy of 1 mg/kg of oral prednisolone can be administered solely under the systemic antiviral therapy, at least 48–72 h later. The tapering is recommended in an average period of 1 month after the start, depending on the clinical findings. In BARN, parenteral or oral steroids without antiviral therapy can develop ocular phthisis [46].

### 3.4. Intravitreal Therapy

Intravitreal antivirals have been proposed in combination with antiviral oral or intravenous therapy, dosing according to the severity of the infection and the patient’s clinical condition. Most reports highlighted using intravitreal foscarnet and ganciclovir as monotherapy or together, also in combination with dexamethasone [47,48,49]. The recommended high intravitreal doses were 2.4 mg foscarnet and 2.0 ganciclovir for the efficacious and tolerable treatment [50].

Intravitreal injections can be administered twice a week at the beginning, and then a frequency reduction to once a week when retinopathies are stable and inactive [38].

A comparative study by Yeh et al. [51] described a significant reduction in the number of months related to the use of intravenous or oral acyclovir or oral valacyclovir in eyes treated with systemic plus intravitreal antivirals compared to eyes that had not been subjected to antiviral injection. They also emphasized a better visual outcome (gains of two lines) and a decreased risk of retinal detachment.

### 3.5. Laser Therapy and Surgery

Panretinal photocoagulation (PRP) is indicated in all cases of extensive retinitis over three-disk areas involved. Two or three rows of lasers are preferable for an effective barrage to prevent rhegmatogenous retinal detachment. Vitrectomy via pars plana is only approved in cases of vitritis with visual disturbance or retinal detachment. It is not recommended as the first step. Post-vitrectomy silicone oil tamponade to repair retinal detachment allows for long-term tamponade in viral retinal necrosis, although complications such as increased intraocular pressure and glaucoma may be associated, and follow-up is mandated [12]. PRP associated with antiviral intravitreal injections is best indicated as the first approach [38].

### 3.6. New Insights on Therapeutic Strategy

Recently, the hypothesis of using biological drugs to treat retinal necrosis from studies in animal models for increased acyclovir-resistant patients is a significant challenge. Bauer et al. [52] explored the efficacy of the humanized monoclonal antibody (mAb) hu2c, which targets HSV-1 and HSV-2 glycoprotein B, in infected mice that have developed acute retinal necrosis resistant to acyclovir treatments. These mice were treated with systemic mAb hu2c either 24 h before infection (prophylactic treatment) or 24, 40, and 56 h after infection (post-exposure treatment). As a result, the control mice that did not have any treatments and those treated with acyclovir showed serious retinal damage, while the mice treated with mAb hu2c did not develop retinal necrosis.

## 4. Conclusions

BARN is a serious ophthalmic condition with often worse visual outcomes if not diagnosed and treated in a timely manner. Serological tests, and especially PCR on aqueous humor or vitreous samples, are key tools for identifying the causative agent, with herpes viruses—in particular, varicella zoster virus (VZV)—remaining the predominant cause. Although VZV is the most frequently identified agent, it is essential to also consider other possible etiologies for a complete and accurate diagnosis. Anatomical and functional damage to the retina tends to progress rapidly.

Despite multimodal imaging and new but still experimental treatments, BARN continues to be very aggressive and associated with poor visual recovery if not diagnosed in time or undertreated.

The only current prevention to avoid blindness and complications still remains the early diagnosis and treatment of the underlying cause. In conclusion, the optimal management of BARN requires immediate and targeted intervention based on accurate clinical and laboratory recognition.

## Figures and Tables

**Table 1:** Acronyms: Polyomavirus—BK virus; Cytomegalovirus—CMV; Epstein–Barr virus—EBV; Herpesvirus6—HHV6; Herpes simplex 1—HSV1; Herpes simplex 2—HSV2; Pseudorabies Virus—PRV; Varicella zoster—VZV. N: number of articles; Type: type of articles. Others: other conditions such as Behcet’s disease, Wegener’s disease, Sarcoidosis, and ocular lymphoma. Corticosteroids *: epidural injection, oral, and intravitreal injection.

Causes	Pathogen or Other Agents	N	Studies
*virus*	VZV	612	case reports, case series, and reviews
	HSV1 and HSV2	214	case reports, case series, and reviews
	CMV	37	case reports, case series, and reviews
	EBV	31	case reports, case series, and reviews
	HHV6	2	case report and review
	PRV	3	case reports
	Coronavirus	3	case reports
	BK virus	2	case reports
*bacterial*	*Treponema pallidum*	23	case reports and reviews
	*Mycobacterium tuberculosis*	2	case series
*parasites*	*Toxoplasma gondii*	68	case reports, case series, and reviews
	*Angiostrongylus cantonensis*	1	case report
*fungal*	*Aspergillus*	6	case series and review
*other*	others	4	case reports
*iatrogen*	Alemtuzumab	1	case report
	Natalizumab	1	case report
	Dapsone	1	case reports
	Corticosteroids *	1	case report
	Lysoform Medical	1	case report

**Table 2:** Acronyms: Polyomavirus—BK virus; Cytomegalovirus—CMV; Epstein–Barr virus—EBV; Herpesvirus6—HHV6; Herpes simplex 1—HSV1; Herpes simplex 2—HSV2; Pseudorabies Virus—PRV; Varicella zoster—VZV; immunocompromised—IC; immunocompetent—IT; viral encephalitis—VE; meningitis—ME; immune deficiency syndrome—IDS; lymphoproliferative disease—LD; systemic lupus erythematosus—SLE; multiple sclerosis—MS, methylprednisolone abuse—METH. Others: other agents not previously mentioned; NA: undetermined. Corticosteroids *: epidural injection, oral, and intravitreal injection.

Pathogens or Other Agents	IC	IT	VE	ME	IDS	LD	SLE	MS	METH	OTHERS	NA
Alemtuzumab	0	1	0	0	0	0	0	1	0	0	0
*Angiostrongylus cantonensis*	1	0	0	1	0	0	0	0	0	0	0
*Aspergillus*	1	0	0	0	1	0	0	0	1	0	0
BK virus	1	0	0	0	0	0	0	0	0	0	0
CMV	1	1	0	0	0	1	1	0	0	0	0
Coronavirus	1	0	0	0	0	1	0	0	0	1	0
Corticosteroids *	0	1	0	0	0	0	0	0	0	0	0
Dapsone	0	1	0	0	0	0	0	0	0	0	0
EBV	1	1	0	0	0	1	0	0	0	0	0
HHV6	0	1	0	0	0	0	0	0	0	0	0
HSV1 and HSV2	1	1	1	0	0	0	0	0	0	0	0
Lysoform medication	0	1	0	0	0	0	0	0	0	0	0
*Mycobacterium tuberculosis*	0	1	0	0	0	0	0	0	0	0	1
Natalizumab	0	1	0	0	0	0	0	1	0	0	0
Ocular lymphoma	1	0	0	0	1	0	0	0	0	0	0
Others	1	1	0	0	0	0	0	0	0	0	1
PRV	1	0	1	0	0	0	0	0	0	0	0
*Toxoplasma gondii*	1	0	0	0	1	0	1	0	0	0	0
*Treponema pallidum*	1	1	0	0	1	0	0	0	0	0	0
VZV	1	1	0	0	1	1	0	0	0	1	0

**Table 3:** Acronyms: Polyomavirus—BK virus; Cytomegalovirus—CMV; Epstein–Barr virus—EBV; Herpesvirus6—HHV6; Herpes simplex 1—HSV1; Herpes simplex 2—HSV2; Pseudorabies Virus—PRV; Varicella zoster—VZV; hand movement—HM; no light perception no-LP; counting fingers CF. Others: other conditions such as Behcet’s disease, Wegener’s disease, Sarcoidosis, and ocular lymphoma. NA: undetermined. Corticosteroids *: epidural injection, oral, and intravitreal injection.

Causes	Pathogens or Other Agents	BCVA	Complications
*virus*	VZV	<20/200-20/40	optic atrophy and retinal detachment
	HSV1 and HSV2	<20/200-20/40	optic atrophy and retinal detachment
	CMV	<20/200	vitreous haze, hemianopia, and diplopia
	EBV	<20/400	thrombosis, macular edema, and retinal detachment
	HHV6	20/60	vitreous haze, hemianopia, and diplopia
	PRV	20/100	retinal detachment
	Coronavirus	NA	vitreous haze and retinal detachment
	BK virus	HM	vitreous haze, vasculitis, and retinal detachment
*bacterial*	*Treponema pallidum*	NA	vitreous haze, vasculitis, and retinal detachment
	*Mycobacterium tuberculosis*	NA	vitreous haze, vasculitis, retinal detachment, and choroidal effusion
*parasites*	*Toxoplasma gondii*	HM, <20/200	aqueous cells, vitreous haze, retinal detachment, and optic atrophy
	*Angiostrongylus cantonensis*	no-LP	vitreous haze and retinal detachment
*fungal*	*Aspergillus*	<20/200	vitreous haze and retinal detachment
*others*	others	<20/200-HM	vitreos haze and sclerosed vessels with perivascular infiltrates
*iatrogen*	Alemtuzumab	20/200	vitreous haze and retinal detachment
	Natalizumab	<20/200-CF	vitreous haze and retinal detachment
	Dapsone	<20/200-CF	optic atrophy, macular edema, retinal detachment, and choroidal effusion
	Corticosteroids *	<20/200	NA
	Lysoform medication	<20/200	NA
